# Analysis of seasonal and long-term population dynamics for modeling populations at low density: Experience with light traps

**DOI:** 10.1371/journal.pone.0354917

**Published:** 2026-08-14

**Authors:** Vladislav Soukhovolsky, Vladimir Dubatolov, Anton Kovalev, Olga Tarasova, Vyacheslav Martemyanov

**Affiliations:** 1 V.N.Sukachev Institute of Forest SB RAS, Akademgorodok, Krasnoyarsk, Russia; 2 Isaev Centre for Forest Ecology and Productivity of the Russian Academy of Sciences (CEPF RAS), Profsoyuznaya, Moscow, Russia; 3 Institute of Systematics and Ecology of Animals of the Siberian Branch of the Russian Academy of Science (ISEA SB RAS), Novosibirsk, Russia; 4 “Zaspovednoe Priamur’e”, Khabarovsk, Russia; 5 Krasnoyarsk Scientific Center of the Siberian Branch of the Russian Academy of Science, Akademgorodok, Krasnoyarsk, Russia; 6 Siberian Federal University, Svobodny Av, Krasnoyarsk, Russia; 7 Research Center for Genetics and Life Sciences, Sirius University of Science and Technology, Olympic Prospect 1, Sirius Federal Territory, Sochi, Russia; University of Mississippi School of Pharmacy, UNITED STATES OF AMERICA

## Abstract

Monitoring and population forecast of major pest species are important tasks for pest management in both agriculture and forestry. In current study we suggest the models for both flight-initiation within a season and multisession population dynamics at low population densities of most important Palearctic folivore species conifer silk moth *Dendrolimus superans* (Lepidoptera: Lasiocampidae). The analysis uses light trap adult catch data collected over 21 years, from 2005 to 2025. Three models of adult flight are considered: a flight-initiation model driven by weather factors, an autoregressive model of long-term catch dynamics, and a binary model of seasonal catch. For the flight-initiation model, we propose estimating the Sum of the Active Temperatures from the date when the first derivative of the Normalized Difference Vegetation Index (NDVI) monitored by remote sensing becomes positive until the date of the first adult capture of the season. Sum of the Active Temperatures is shown to be sufficiently stable across all years of observation, with flight each year beginning after this temperature sum is reached. The second model demonstrates that the long-term light trap catch time series is well described by a second-order autoregressive model AR(2), in which the catch of the current year depends on catches from the two preceding years. This long-term series is compared with a previously studied larval population density series of the Siberian silk moth; both are shown to be AR(2) series with similar coefficient values, which suggesting that adult catch data may serve as a proxy for absolute larval population density. In the third model, we describe the transition from absolute-scale seasonal catch dynamics (number of adults per day) to a binary scale (0, 1), where 0 denotes days on which no adults were attracted to the trap, and 1 denotes days on which at least one individual was captured.

## Introduction

Forecasting population outbreaks of eruptive pest species is an important goal of both fundamental and applied ecology [1,2]. One productive approach to ecological insect monitoring is pheromone trapping, which, with relatively modest observer effort, provides a quantitative estimate of the current population density of the target species [3]. An alternative is light trapping [4]; however, this approach requires additional expertise in entomological taxonomy, since light traps for nocturnal insects (e.g., moths) have lower species specificity. This limitation is increasingly being offset by the rapid development of artificial intelligence and deep learning methods for species identification [5,6]. One of the pressing challenges in using any type of observation, including light trapping, is the low information content of time series (a lot of “zero” observations) when analyzing population density during the post-outbreak decline phase, or in stably sparse populations [7].

In our study, we assembled a 21-year time series of population density observations for *Dendrolimus superans* to characterize its population in the Russian Far East. *D. superans* (Lepidoptera: Lasiocampidae) is a serious pest of coniferous forests in the Russian Far East and northern China [8–10]. For many years it was believed that the principal damage in Siberian forests was caused by the subspecies *D. superans sibiricus* Tschetv. However, recent genetic analysis has shown that Far Eastern populations of *D. superans* form a distinct cluster, and *D. sibiricus* might now recognized as a separate species [11]. The phenotypic differences between these groups are not strongly pronounced and some authors still regard them as subspecies [12]. Both *D. superans sibiricus* and *D. superans superans* may occur in the Far Eastern region. Our goal was not to assess *D.* speciation, which, based on genetic evidence, is most probably ongoing [11,13,14]; we therefore do not focus on this question in the present study, noting only that in all likelihood basing on range location we were working with a population of *D. superans* inhabiting the area closer to the Pacific coast.

Interest in studying members of the genus *Dendrolimus* is driven by two concerns: the risk of irreversible forest damage within the species’ range [15,16] and the risk of invasion into new territories [13,17,18]. Given the potential risks associated with introduction of this pest into European countries, it has been included in the A2 quarantine list (i.e., present, but not yet causing economic damage) of EPPO (European and Mediterranean Plant Protection Organization), and stringent regulations are enforced to prevent its inadvertent introduction [19]. Despite the economic importance of this pest to forestry on a global scale, information on the long-term population dynamics of this species is extremely limited. Only two sufficiently long datasets are known, presented in [8] and [20]. The scarcity of available data on such an important pest stems from two difficulties in population censusing. First, monitoring typically begins when the pest enters the outbreak phase and, by damaging trees, effectively marks the zone where reliable counts can be conducted. Second, once the outbreak ends, the pest population density declines markedly, and the appropriate location for further surveys becomes unclear. In the above-cited studies [8,20], surveys were conducted across a sufficiently large territory, and the published data can be regarded as integrated population characteristics. Long-term monitoring of pest populations in a stably sparse state (i.e., when population density is insufficient to cause visible defoliation) is problematic, since it is unclear where to conduct surveys and what the practical significance of such monitoring would be in a territory where outbreaks may never occur. However, studying pest population dynamics at low density may yield important information about the regulatory mechanisms of the species.

Since no stand damage occurs at low population density, damage data cannot be used to select survey sites, and locating larvae or pupae across vast taiga forest areas is impractical. Only one method can be proposed for studying the long-term dynamics of the Siberian silk moth at low density: long-term monitoring of adult catches using pheromone or light traps.

If a light trap is regarded as a tool for estimating pest population density, there are two approaches to measuring trap catch: cumulative and differential. In the cumulative approach, the trap is placed at a site at time t_0_, approximately before the onset of adult flight, and the total catch is counted at approximately the time of flight cessation t_f_. This case flight phenology will be missed. In the differential approach, the trap is placed at time t_0_ and catch is recorded at intervals of every m days (at the limit, m = 1, i.e., daily catch is recorded). The differential method is considerably more labor-intensive; however, whether the additional information on population state justifies this increased effort remains an open question. In the present study, data on adult catches by light trap at a local site with coordinates 48.2985°N, 134.8222°E in the forests of the Russian Far East about 20 km west from Khabarovsk from 2005 to 2025 were used to analyze seasonal adult flight dynamics and long-term population dynamics of *D. superans*.

## Materials and methods

### Ground-based adult flight monitoring

**Fig 1:**
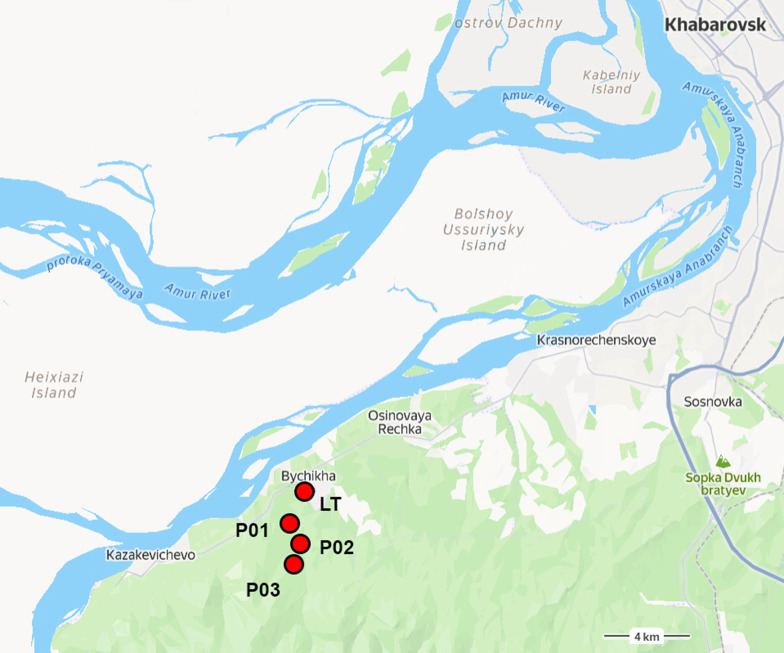
Map of the study area (location coordinates is presented in Table 1 with same localities names).

The monitoring was carried out in the Russian Far East (Fig 1, Table 1).

**Table 1:** Location of the light trap and conifer (host plant) stands P01–P03 near the village of Bychiha (Russian Far East) as sources of Siberian silk moth adults.

Study plot	Coordinates		Distance from trap to plot (m)
	Latitude (°N)	Longitude (°E)	
Light trap (LT)	48.2985	134.8222	0
P01	48.2838	134.8127	1790
P02	48.2766	134.8169	2480
P03	48.2706	134.8166	3145

### Materials and methods

Ground-based moth flight monitoring of *D. superans* was conducted at the outskirts of the village of Bychiha (48.2985°N; 134.8222°E) at the edge of a mixed coniferous–broadleaved forest. A mercury-quartz fluorescent DRV lamp (160 W, luminous flux 2300 lm) during night time. The biology of *D. superans* is briefly described here, and is closely resembles that of *D. sibiricus*. *D. superans* is a conifer-feeding folivorous Lepidoptera, capable of mass population outbreaks. Adult flight begins in June. After mating females lay eggs on the needles of host tree (conifer species, such as fir). Newly hatched larvae feed on the needles of host plant until autumn and then overwintering as mid-instar larvae in the forest litter [21]. After overwintering larvae continue to feed in spring and pupate in the middle of June. Adult flight in July completes the lifecycle. Thus, at high population densities, larvae can cause severe defoliation of host trees.

### Assessment of stand condition and potential tree damage

To assess potential foliage removal by insects, the seasonal dynamics of the photosynthetic vegetation index NDVI were examined. It is known that when trees are damaged by folivorous insects, both the seasonal NDVI sum and the seasonal NDVI maximum decrease [22]. Accordingly, changes in the seasonal NDVI curve can serve as an indicator of substantial damage levels. Seasonal NDVI data were obtained from the MODIS AQUA/TERRA satellite system operating under NASA’s Earth Observing System (EOS) program [23]. These data were used to calculate derived indices: annual NDVI sum and annual NDVI maximum.

### Model for the onset of adult flight

We propose estimating the conditions at which adult flight to the light trap begins following the approach used to assess phenological phase duration in trees.

For trees, the standard method for assessing the Sum of the Active Temperatures (SAT) required for the start of leaf budding includes determining the date t_0_ when the air temperature T exceeds the critical value T_0_ (usually +5 ^0^C), determining the date t_1_ of the start of leaf budding based on phenological observations, and calculating the accumulatedtemperature ${\text{SAT(t}}_{\text{0}},t{\;}_{\text{1}}\;)=\sum_{{\text{t}}_{\text{0}}}^{{\text{t}}_{\text{1}}}\text{T(t)}$ based on meteorological data.

Theoretically, the accumulated temperature SAT(t_0_, t_1_) should be constant (or at least fluctuate slightly without a significant temporal trend) across all years of observation [24–26]. It can then be considered an indicator of the seasonal dynamics of tree development.

A similar approach can be used to estimate the Sum of the Active Temperatures for Insects (SATI) required for adult insects to begin flight. Three parameters are required to calculate the SATI: the date t_0_ when the temperature crosses the critical value T_0_, the date t_1_ of the first capture of adult individuals, and daily air temperature data for the intervening period. Phenological studies typically rely on data from weather stations; however, given the very sparse network of stations in the Asian part of Russia, the nearest station to the observation site may be more than 100 km away, making such data unrepresentative. Therefore, daily satellite data on land surface temperature (LST), which closely reflect air temperature, were used to estimate local air temperature in the study area [27]. The pixel size of the LST satellite data is 1 × 1 km, which is sufficient for a reliable assessment of local weather conditions.

### Autoregressive model of long-term catch dynamics

The authors have previously shown that an autoregressive model AR(k) can be used to describe long-term insect population dynamics, where the current population density depends on densities from the preceding k seasons [28]. To reduce random errors, high-frequency filtering was applied using the Hann filter:


$$\begin{array}{c} \text{NF(i)}=\text{0.24}\cdot \text{N(i-1)}+\text{0.52N(i)}+\text{0.24N(i}+\text{1)} \end{array}$$ (1)

Assuming the long-term dynamics series is described by an autoregressive model of equation (1), the order k of the AR(k) model must first be determined. The standard approach is to calculate the partial autocorrelation function (PACF) [29]. The autoregressive order is defined as the value of k at which the PACF falls within its standard error bounds.

In general form, the AR(k) model for long-term catch dynamics is expressed as:


$$\begin{array}{c} \text{ln N(i)}={\text{a}}_{\text{0}}+\sum_{j=1}^{\text{k}}a_{i-j}ln\;N(i-j) \end{array}$$ (2)

If the order of autoregression and the values of the time series {N(i)} in equation (1) are known, equation (1) can be treated as a linear regression equation with unknown parameters a₀, …, aₖ, whose values can be estimated using standard linear regression methods [30].

### Binary model of seasonal catch dynamics

The number of adult individuals caught in the light trap was recorded daily. Flight events during the season can be divided into two types. **Regular** flight is characterized by a nonlinear unimodal temporal dependence of flight intensity, with daily catch substantially greater than zero. **Sparse** flight is characterized by the absence of daily adult captures and low absolute catch – the total number of adult Siberian silkmoth caught in the trap.

To describe flight dynamics as a set of rare events, we convert the absolute daily catch scale (number of adults per day) to a binary scale, where 0 denotes days on which no adults were attracted to the trap, and 1 denotes days on which captures occurred. Flight dynamics are thereby represented as a binary series of zeros and ones.

For such a series, the presence of a temporal trend – manifested as a change in the probability of observing state 0 over time – can be assessed. Let’s define p(0) as the probability that there is no catch in the trap.

If for different time segments p(0) = const, the binary time series can be regarded as stationary with probability p(0) of observing state 0 and probability p(1) of observing state 1.

A key question is whether the binary series is independent (i.e., there are no correlations between the values of p(0) at arbitrary points in time), and if not, whether the probability p(0, j) at time j depends on the state of the series at times (j-1), (j-2), … (j-k).

In the simplest case, where the state of the series at time j depends on its state at time (j − 1), we are dealing with a Markov chain [31].

Testing the Markov properties of the series is straightforward: the numbers of event pairs (0,0), (0,1), (1,0), and (1,1) are calculated for the series under study, and these are compared with the expected counts [p(k)×p(r)×N] using the χ²-test, where p(k) and p(r) are the proportions of events with values k = 0,…1 and r = 0,…1, and N is the length of the time series. If the χ²-test value is less than the tabular critical value $\chi_{c}^{\text{2}}\left(n\right)$ for the given degrees of freedom n, the observed series can be said to lack Markov properties, and the current value of the series is independent of the preceding value.

## Results

### Flight-initiation model

Since there is no theoretical basis for selecting the reference date t₀ from which accumulated temperature is calculated, t₀ was determined from seasonal NDVI dynamics. Two time markers were proposed: the first reference point t₀ – the date when NDVI begins to rise in spring and the difference between successive NDVI values becomes positive; the second point t₁ – the day when the first adult is found in the light trap. The accumulated temperature sums SATI between points t₀ and t₁ at the trap location should be constant across years for a given species following the same logic as heat sums used for phenological phases.

**Fig 2:**
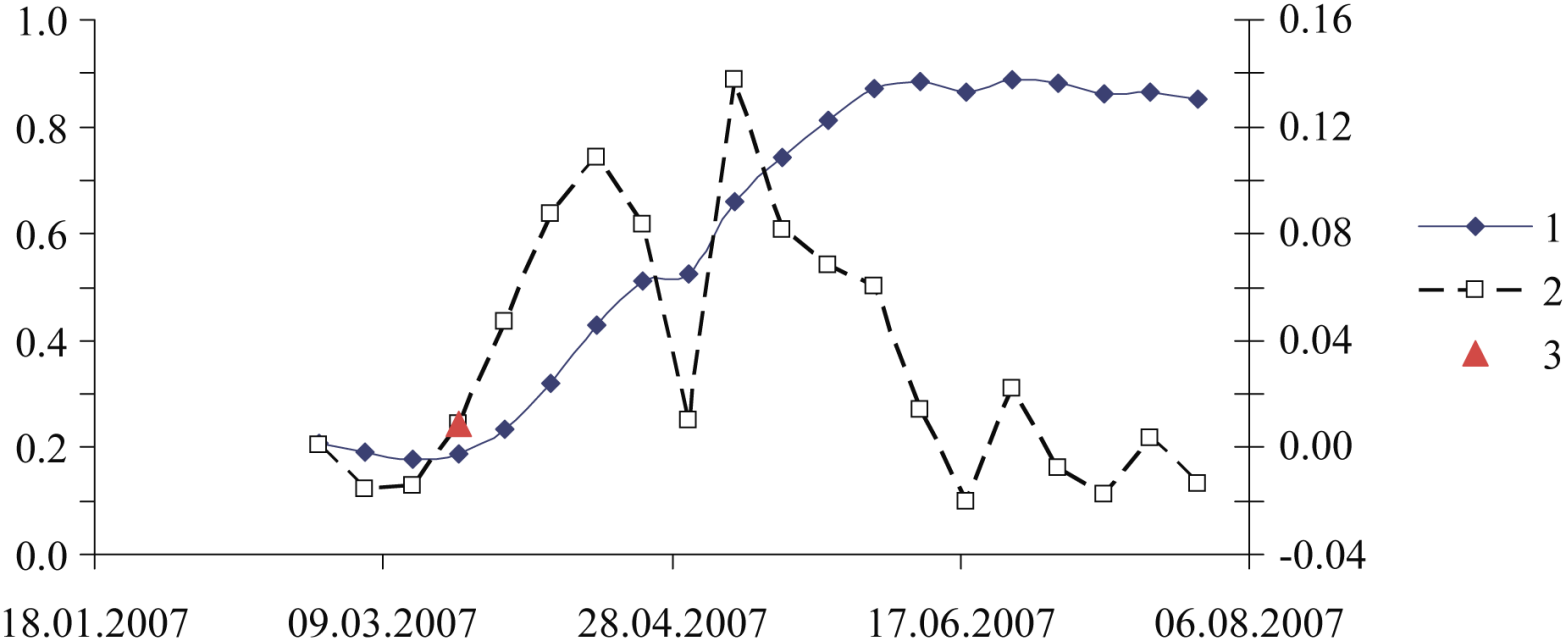
Seasonal dynamics of the NDVI series values (1), the first-difference of NDVI series (2), and the critical time point (3) for series (2).

For the proposed method, the critical points t₀ and t₁ were determined (typical seasonal NDVI curves and first-difference NDVI series are presented in Fig 2).

For each year, the logarithm of the daily LST sum from date t₀ to date t₁ was calculated (Table S1 in S1 File the Supplement). Calculations showed that the values of SATI(i) for all i (i = 1, …, 21) years are very similar: the long-term mean SATI equals 7.38, with a standard deviation s = 0.23 and standard error sₓ = 0.054. The variability of SATI(i) among individual years is therefore very small, and flight begins each year after the accumulation of an approximately constant temperature sum. Thus, it can be said that the SATI values for different years are quite similar and serve as a threshold condition for the start of flight.

### Long-term catch dynamics model

**Fig 3:**
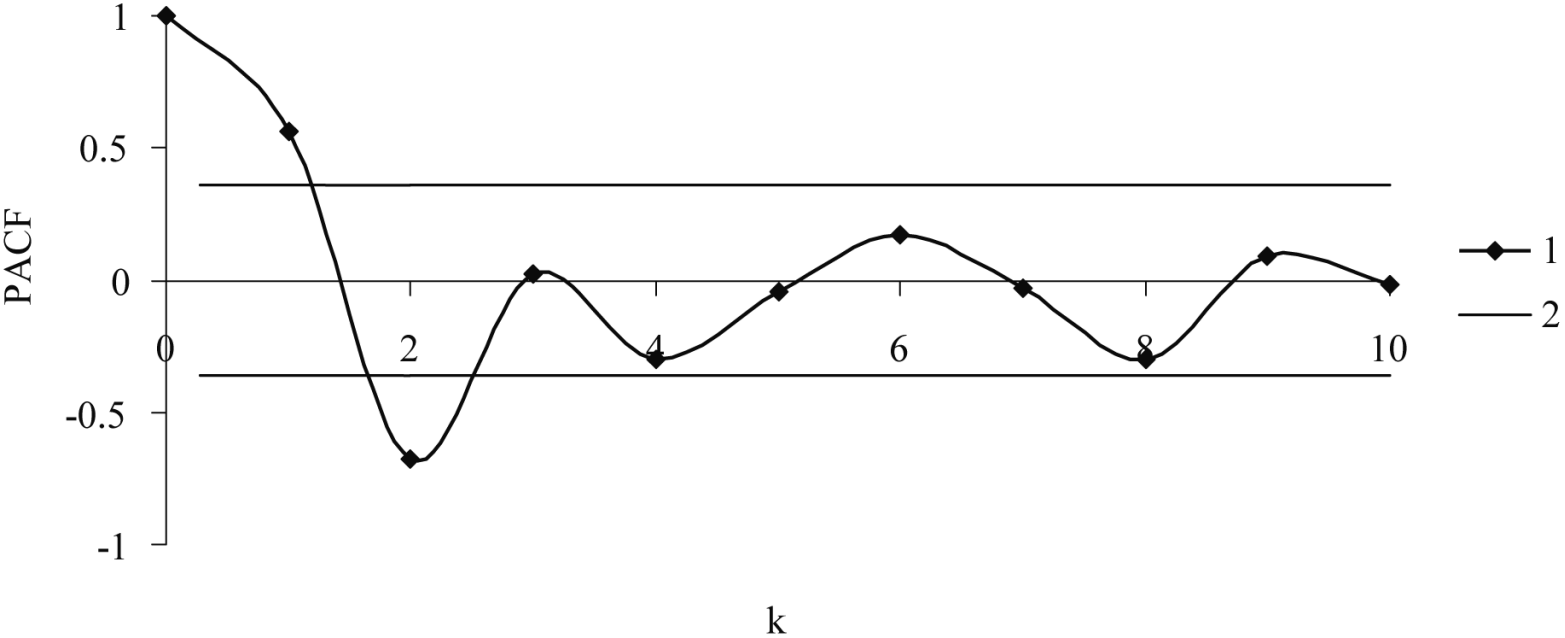
Partial autocorrelation function (PACF) of the long-term catch dynamics series. 1 – PACF; 2 – std. error of PACF.

To determine the order of the autocorrelation model, the PACF is calculated. The PACF for the long-term adult catch series of the Siberian silk moth is shown in Fig 3.

As shown in Fig 3, the PACF value is not significantly different from 0 (it does not fall outside the standard error band) for time shift values k > 2, this means that the autoregressive order k equals 2 for the catch series.

The AR(2) model coefficients are given in Table 2.

**Table 2:** AR(2) model for the catch series.

Parameter	Coeff.	Std. Err.	t-test	p-value
a0	14.415	4.558	3.162	0.008183
N(i-2)	−0.732	0.196	−3.732	0.002863
N(i-1)	0.997	0.199	5.018	0.000300
Adj R²	0.630			
F-test	13.14			

**Fig 4:**
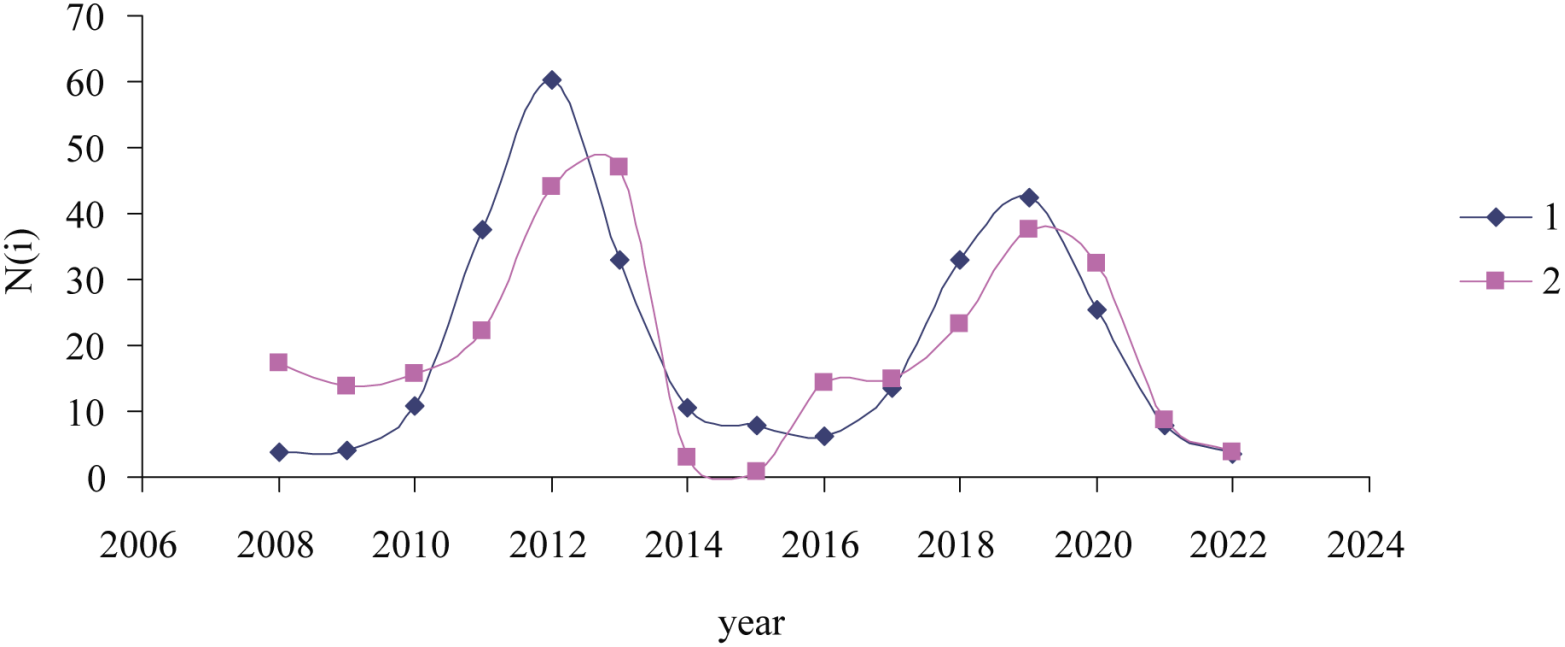
Long-term catch dynamics of the Siberian silk moth after high-frequency filtering with the Hann filter. 1 – observed data; 2 – AR model.

Fig 4 shows the time series of seasonal catch N(i) over the study period.

As seen in Table 2, the current light trap catch is influenced by the catches of seasons (i-1) and (i-2). The coefficient a₁ is positive and a₂ is negative. These coefficients describe how current catch is regulated by catches from the two preceding years. The same signs of the feedback coefficients are characteristic of population dynamics series for the Siberian silk moth and, more generally, of a large number of forest insect species exhibiting outbreak dynamics [32]. As can be seen in Fig 4, the long-term trend in the flight of adult moths to the light trap is cyclical. For comparison, the larval density series for the Siberian silk moth in the Russian Far East is described by the model equations in Table 3.

**Table 3:** AR(2) model coefficients for the larval density series x(i) of the Siberian silk moth in the Russian Far East.

Parameter	Value	Std. Err.	t-test	p-value
a0	−0.448	0.214	−2.095	0.051499
Ln x(i-2)	−0.767	0.125	−6.154	0.000011
Ln x(i-1)	1.381	0.121	11.380	0.000000
Adj R²				
F-test				

**Fig 5:**
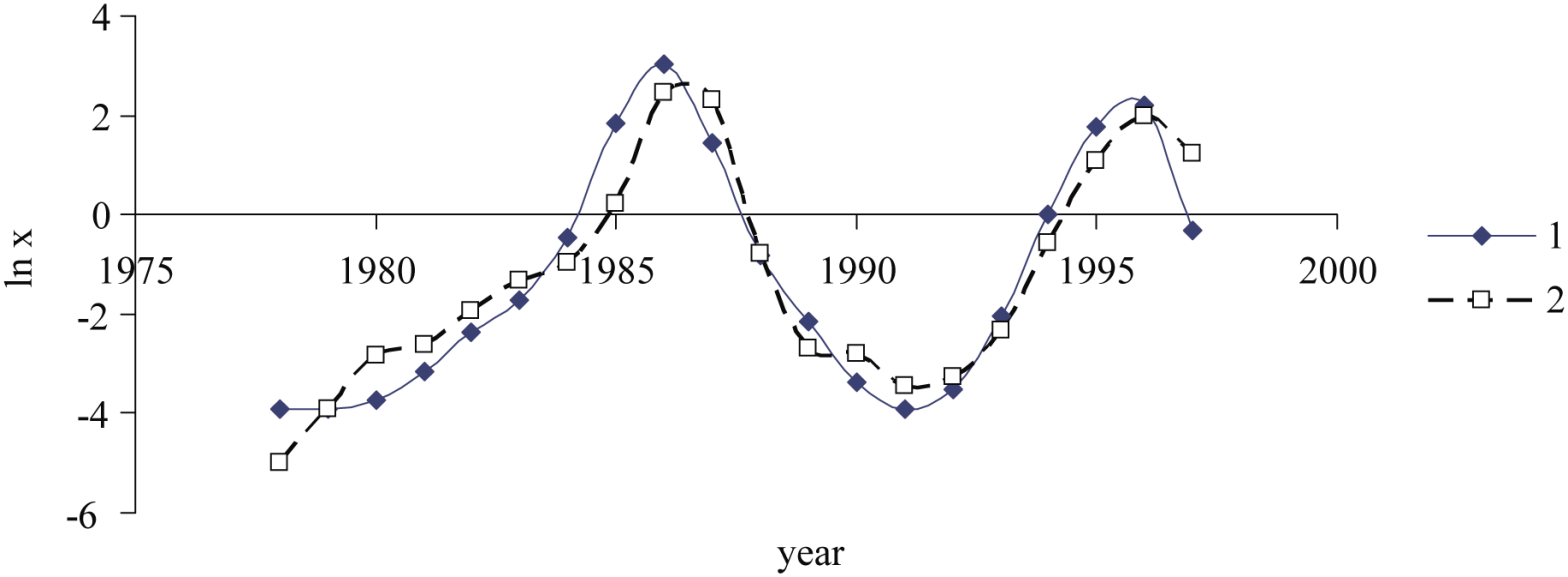
Population dynamics of the Siberian silk moth in the Russian Far East. 1 – observed data [20]; 2 – AR(2) model.

Fig 5 shows field observation data from [20] and the modeled AR(2) population density series for the Siberian silk moth in Khabarovsk suburbs.

Comparing Figs 4 and 5, it can be concluded that although the series differ in developmental stage assessed (larvae vs. adults) and the analyzed period (adults: 2005–2025; larvae: 1978–1997), the model coefficients are very similar. This indicates that the oscillation periods of the larval and adult count time series are comparable.

To assess the impact of weather and stand condition on the summer adult emergence of the Siberian silkmoth, the relationship between catch rates and the average LST during the summer period—as an indicator of weather conditions—and the NDVI response to changes in LST was examined. The correlation coefficient between the mean seasonal LST and light trap catch equals 0.03, indicating no significant relationship between weather during the flight season and trap catch.

### Stand condition and damage risk assessment

When estimating larval pest population density, the risk of stand damage can be assessed if the individual consumption rate is known. However, light trap data do not allow direct estimation of absolute larval population density. The coefficient H linking seasonal light trap catch to larval population density is likewise unknown. To assess the risk of stand damage, it is therefore necessary to evaluate the relationship between total seasonal catch and the level of pest impact on the stand, as characterized by the seasonal NDVI sum and NDVI maximum. An analysis of NDVI data from years with low catch rates of adult Siberian silkworms showed that, in the absence of damage, the seasonal NDVI sum should be close to 20 and the NDVI maximum close to 1.

Table S2 in S1 File presents long-term dynamics of the NDVI sum and maximum at study plot P01 from weeks 13–41. As shown in Table S2 in S1 File, the calculated NDVI values did not change significantly throughout the entire study period, indicating the absence of severe foliage damage by the pest. This is corroborated by our long-term field observations. Similar results were obtained for study plots P02–P05. It can therefore be concluded that no sharp increase in Siberian silk moth abundance occurred at any point during the study period, and the population remained in a stably sparse state throughout the study period.

### Binary catch model

Since catch rates vary widely, a transition was made from absolute catch values to a binary scale to reduce random fluctuations in catch rates, where a value of 1 corresponds to a day when adults were caught in the trap, and a value of 0 corresponds to a day when the catch was zero. Table 4 presents light trap catch data for 2018 on both the absolute and binary scales.

**Table 4:** Absolute and binary light trap catches in 2018.

Date	Absolute catch	Binary catch	Date	Absolute catch	Binary catch
01.07.2018	4	1	16.07.2018	2	1
02.07.2018	1	1	17.07.2018	3	1
03.07.2018	0	0	18.07.2018	0	0
04.07.2018	1	1	19.07.2018	3	1
05.07.2018	0	0	20.07.2018	0	0
06.07.2018	0	0	21.07.2018	0	0
07.07.2018	0	0	22.07.2018	4	1
08.07.2018	1	1	23.07.2018	0	0
09.07.2018	0	0	24.07.2018	0	0
10.07.2018	2	1	25.07.2018	2	1
11.07.2018	1	1	26.07.2018	1	1
12.07.2018	0	0	27.07.2018	0	0
13.07.2018	0	0	28.07.2018	0	0
14.07.2018	0	0	29.07.2018	3	1
15.07.2018	3	1	30.07.2018	0	0
			31.07.2018	2	1

33 adults were captured over 31 flight days (Table 4), and the probabilities p(0) = 0.516 and p(1) = 0.484 can be readily calculated.

Table 5 presents the numbers of event pairs (0,0), (0,1), (1,0), and (1,1) for the observed binary series and for the independent series.

**Table 5:** Numbers of event pairs (0,0), (0,1), (1,0), and (1,1) for the observed binary series and for the independent series.

Binary variable value	Series type	Binary variable value	
		0	1
0	Observed	7	9
	Independent	7.99	7.49
1	Observed	9	5
	Independent	7.49	7.02

From the data in Table 5, the χ²-test value can be calculated as:


$$\begin{array}{c} \chi^{\text{2}}=\sum \frac{{\text{(n(k,r)-m(k,r))}}^{\text{2}}}{\text{m(k,r)}} \end{array}$$ (3)

where n(k,r) is the observed number of pairs with k and r equal to 0 or 1, and m(k,r) is the expected count under the independence model.

For the data in Table 5, χ² = 1.31, which is substantially less than the critical value χ²(cr) = 3.84 at p = 0.05. Adult flight to the light trap in 2018 can therefore be regarded as a random process with approximately equal probabilities of capture and non-capture on any given day.

Analogous data for 2019 are presented in Tables S3 and S4 in S1 File, and for 2020 in Tables S5 and S6 in S1 File. In 2019, 55 individuals were captured, p(1) = 0.55, p(0) = 0.45, and χ² = 0.48, indicating that connections between adjacent values in the binary series are insignificant (p = 0.49 и 0.42). In 2020, 21 individuals were captured, p(1) = p(0) = 0.50, and χ² = 0.65, which indicates the independence of adjacent values in the contingency Table S6 in S1 File.

As flight transitions from sparse random to regular, p(1) → 1. In the case of very sparse random flight, p(0) → 1 (i.e., no individuals were captured). Thus, flight intensity can be estimated based on the parameters p(1) and p(0). For the 2018–2020 data, p(1) ≈ p(0) ≈ 0.50, indicating that the flight intensity was approximately the same across all three years.

**Fig 6:**
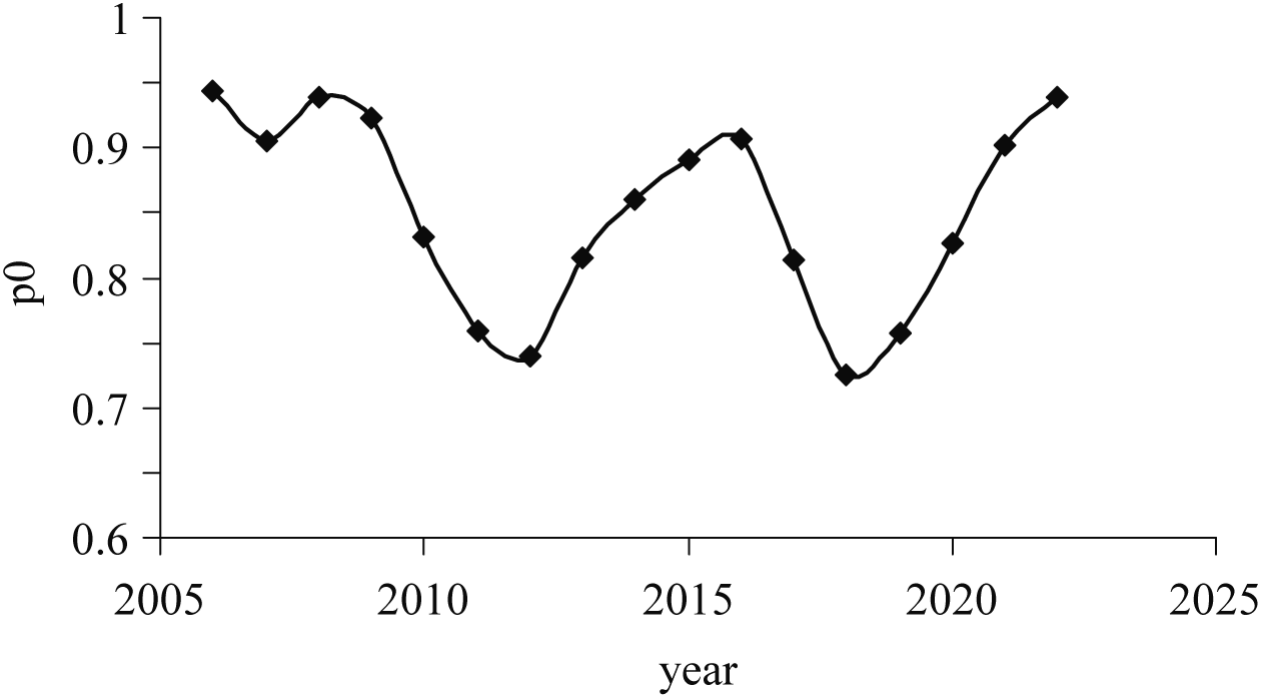
Temporal dynamics of the p₁ series.

Analogously to the long-term absolute catch dynamics series (Fig 4), an AR(2) model of the p₁ series can be constructed (Fig 6).

The order of the PACF of the p₁ series equals 2; the estimated AR(2) model coefficients for the binary catch time series are presented in Table 6.

**Table 6:** AR(2) model coefficients for the variable p₁.

Parameter	Value	Std. Err.	t-test	p-value
h0	0.496049	0.142827	3.47308	0.004605
p0(i-2)	−0.733549	0.193838	−3.78434	0.002603
p0(i-1)	1.147854	0.202945	5.65598	0.000106
Adj R²	0.680000			
F-test	16.020000			

**Fig 7:**
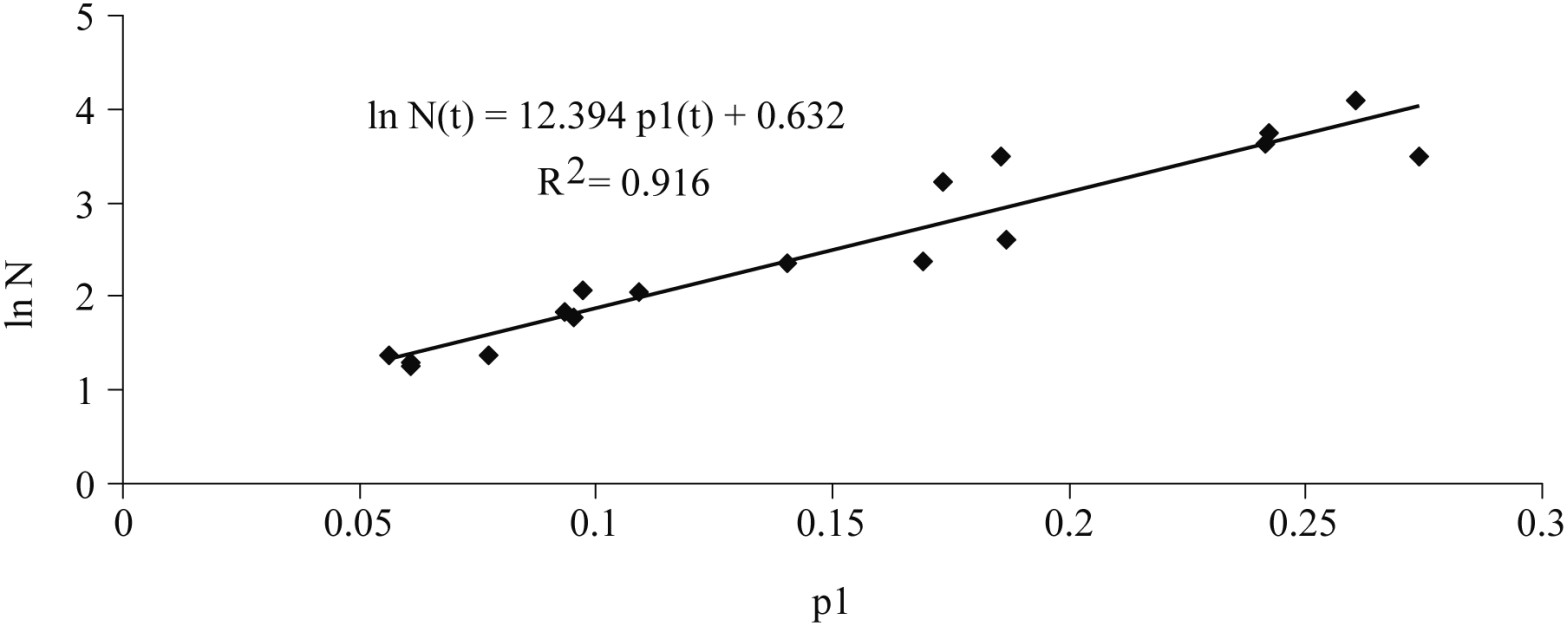
Relationship between p₁(t) and ln N(t).

The absolute and binary catch series are in phase, and binary catch values can be used in place of absolute catch measurements (Fig 7).

As shown in Fig 7, knowledge of p₁ allows estimation of the absolute catch N.

## Discussion

When studying the long-term population dynamics of pest species in which periodic outbreaks lead to sharp increases in population density, the long-term dynamics curve is often cyclic. However, in most cases it remains unclear how population dynamics behave during the chronic low-density (inter-outbreak) phase, when damage foci are absent. Two types of dynamics are possible: random fluctuations around a low mean value, or cyclic oscillations similar to those during outbreaks but with a smaller amplitude. Our ground-based and remote sensing observations of the *D. superans* in the Russian Far East revealed a prolonged (longer than the average interval between outbreaks—7 years) depression of this species in the region, consistent with data from other researchers obtained during the same period [16]. This was confirmed both by light attraction monitoring and by satellite data analyzed using the remote sensing approaches developed in our earlier work [22]. The interval between population peaks was approximately 7 years, consistent with the periodicity of populations reaching peak densities [33]. Conditions in the Far East apparently do not favor the full expression of the Siberian silk moth’s outbreak potential. One possible reason is the relatively high tree species diversity in Far Eastern forests compared with the continental Siberian taiga. Being a pronounced oligophage [34], *D. superans* is limited in its choice of a diverse resource, fully expressing its biotic potential and reaching high population densities.

Comparison of the model parameters in Table 2 with those of the model previously developed for long-term *D. superans* dynamics in the forests of Khabarovsk Krai [35] shows that both outbreak populations and populations at low density are governed by the same system of positive and negative feedback. An outbreak may therefore not involve the emergence of a new regulatory mechanism but rather reflect, for example, a change in the state or availability of food resources [36]. It can accordingly be hypothesized that at low population density, regulatory processes operate through the same mechanism that governs fluctuations during outbreaks.

The use of a binary catch scale made it possible to take zero-catch results into account. After accounting for accumulated temperature sums, flight onset timing was shown to be directly governed by ambient temperature conditions. The Siberian silk moth exhibits ontogenetic variability. Usually, eggs are laid on host tree needles, and first-instar larvae feed for about 3–4 weeks. By September, second or early third instar larvae descend to the forest litter and overwinter. Larvae resume feeding the following spring, continue developing, pupate and emerge mainly in June – first half of July. This represents a rapid life cycle [37,38]. However, under some environmental conditions, a subset of larvae experiences an extended larval stage, prolonging development through the summer months. These larvae descend again in autumn for a second overwintering, thus exhibiting a three-season life cycle [38]. This ontogenetic variability may cause shifts in adult flight timing between different cohorts; however, the long-term data from the south of continental Russian Far East show that the primary driver of variation in flight onset timing is ambient temperature. The mathematical result obtained from a substantial sample of seasonal observations demonstrates the applicability of the proposed model to data from sparse populations, where large numbers of zero-value observations are inevitable. This approach resolves the problem of catch variability at low insect population density.

The ecological causes of flight as a rare event primarily reflect low insect density in the study area, although poor propagation of the light signal cannot be excluded, since light travels in straight lines and landscape elements may obstruct its interaction with moths (scattering, absorption, etc.). In such cases, it is advisable to install multiple traps and to assess the variability of p(1) across them. If the maximum p(1) values for a group of traps are low, this most likely indicates low population density in the area. A trap network may also help delineate the boundaries of an outbreak zone. Another cause of intermittent flight may be weather during the flight period; however, at high population density, catches on days favorable for flight will be correspondingly high.

Our study demonstrates that long-term sparse populations of one of the most important phytophages of boreal forests follow the same ecological dynamics as eruptive populations of this or closely related species elsewhere in the range. Understanding the reasons why outbreak potential is constrained in specific regions will provide a foundation for pest population management. Our study also empirically demonstrated, using a large sample, the applicability of binary models to time series containing large numbers of zeros. This enables analysis of population dynamics data for species that spend far more time at low density than in an eruptive phase.

A comparison of catch rates on absolute and binary scales revealed that these scales are interrelated (see Fig 7) and that the catch cycle is the same on both scales. This suggests that the simpler, less noisy binary scale is preferable for analyzing catch rates and identifying periods of pest outbreaks. The analysis showed that the flight dynamics of Siberian silkmoth adults and their variability across years are consistent with larval counts of this species in the Russian Far East. Fluctuations in adult catch rates and larval numbers are consistent, suggesting that a simpler method of light trap counts based on binary catch rate data can be used to assess pest population dynamics. Further development of light trap counting methods could involve the use of a network of traps placed spatially and comparing correlations between catches in individual traps. If all coefficients of the catchability correlation matrix are close to 1, this may indicate a common source of adult flight. If the coefficients of this correlation matrix differ, this may indicate the existence of multiple sources of Siberian silkmoth flight.

## Supporting information

S1 File Additional data on trapping monitoring and phenological analysis of the Siberian silk moth *Dendrolimus superans* population in the Russian Far East. (DOC)
