# Supplementary material for: Analysis of seasonal and long-term population dynamics for modeling populations at low density: Experience with light traps
Source: PLoS One. 2026 Aug 14;21(8):e0354917. doi: 10.1371/journal.pone.0354917 (PMC13475900; doi:10.1371/journal.pone.0354917)
Supplement: Supplementary file 1 [file pone.0354917.s001.doc]

Supplement

Additional data on trapping monitoring and phenological analysis of the Siberian silk moth *Dendrolimus superans* population in the Russian Far East.

S1 Table. Accumulation start dates and moth flight onset dates, logarithm of the sum of temperatures and cumulative light trap catches at plot LT.

| Year | Accum. start date | Flight onset date | Log. temp. sum | Cum. catch (ind.) | Year | Accum. start date | Flight onset date | Log. temp. sum | Cum. catch (ind.) |
| --- | --- | --- | --- | --- | --- | --- | --- | --- | --- |
| 2005 | 15.04 | 11.7 | 7.32 | 1 | 2016 | 21.3 | 29.06 | 7.43 | 5 |
| 2006 | 15.04 | 25.06 | 7.25 | 3 | 2017 | 14.03 | 7.06 | 7.19 | 8 |
| 2007 | 7.04 | 11.07 | 7.56 | 9 | 2018 | 22.03 | 2.06 | 7.15 | 34 |
| 2008 | 6.04 | 24.06 | 7.36 | 2 | 2019 | 22.03 | 10.06 | 7.31 | 56 |
| 2009 | 30.03 | 5.07 | 7.54 | 2 | 2020 | 29.03 | 13.07 | 7.63 | 21 |
| 2010 | 30.03 | 11.08 | 7.94 | 10 | 2021 | 14.03 | 18.06 | 7.41 | 4 |
| 2011 | 30.03 | 17.07 | 7.63 | 21 | 2022 | 30.03 | 7.06 | 6.98 | 3 |
| 2012 | 29.03 | 2.06 | 6.96 | 101 | 2023 | 14.03 | 21.06 | 7.42 | 4 |
| 2013 | 7.04 | 12.07 | 7.54 | 11 | 2024 |  |  | - | 0 |
| 2014 | 14.03 | 3.06 | 7.27 | 12 | 2025 |  |  |  | 0 |
| 2015 | 30.03 | 30.06 | 7.37 | 7 |  |  |  |  |  |

S2 Table. Long-term dynamics of NDVI sum and maximum by remote sensing data at study plot P1.

| Year | NDVI sum | NDVI maximum | Year | NDVI sum | NDVI maximum |
| --- | --- | --- | --- | --- | --- |
| 2005 | 19.095 | 0.922 | 2016 | 21.495 | 0.959 |
| 2006 | 19.002 | 0.929 | 2017 | 20.812 | 0.925 |
| 2007 | 20.436 | 0.887 | 2018 | 19.743 | 0.948 |
| 2008 | 20.498 | 0.899 | 2019 | 18.22 | 0.963 |
| 2009 | 18.285 | 0.931 | 2020 | 19.464 | 0.92 |
| 2010 | 19.828 | 0.918 | 2021 | 21.336 | 0.915 |
| 2011 | 19.532 | 0.946 | 2022 | 19.483 | 0.924 |
| 2012 | 19.716 | 0.944 | 2023 | 21.043 | 0.93 |
| 2013 | 19.215 | 0.935 | 2024 | 20.252 | 0.917 |
| 2014 | 19.966 | 0.921 | 2025 | 21.101 | 0.9242 |
| 2015 | 21.014 | 0.93 |  |  |  |
| Long-term mean | 19.978 | 0.928 | Std. error | 0.207 | 0.0039 |

S3 Table. Absolute and binary catches in 2019 at plot LT.

| Date | Absolute catch | Binary catch | Date | Absolute catch | Binary catch |
| --- | --- | --- | --- | --- | --- |
| 07.07.2019 | 1 | 1 | 17.07.2019 | 7 | 1 |
| 08.07.2019 | 0 | 0 | 18.07.2019 | 4 | 1 |
| 09.07.2019 | 2 | 1 | 19.07.2019 | 0 | 0 |
| 10.07.2019 | 0 | 0 | 20.07.2019 | 0 | 0 |
| 11.07.2019 | 0 | 0 | 21.07.2019 | 0 | 0 |
| 12.07.2019 | 0 | 0 | 22.07.2019 | 18 | 1 |
| 13.07.2019 | 1 | 1 | 23.07.2019 | 9 | 1 |
| 14.07.2019 | 1 | 1 | 24.07.2019 | 0 | 0 |
| 15.07.2019 | 0 | 0 | 25.07.2019 | 8 | 1 |
| 16.07.2019 | 1 | 1 | 26.07.2019 | 3 | 1 |

S4 Table. Numbers of event pairs (0,0), (0,1), (1,0), and (1,1) for the observed binary series in 2019 and the independent series at plot LT.

| Binary variable value | Series type  Observed | Binary variable value | |
| --- | --- | --- | --- |
|  |  | 0 | 1 |
| 0 | Independent | 4 | 6 |
|  | Observed | 3.85 | 4.70 |
| 1 | Independent | 5 | 5 |
|  | Series type | 4.7025 | 5.7475 |

S5 Table. Absolute and binary catches in 2020 at plot LT.

| Date | Absolute catch | Binary catch | Date | Absolute catch | Binary catch |
| --- | --- | --- | --- | --- | --- |
| 13.07.2020 | 1 | 1 | 22.07.2020 | 1 | 1 |
| 14.07.2020 | 0 | 0 | 23.07.2020 | 0 | 0 |
| 15.07.2020 | 0 | 0 | 24.07.2020 | 3 | 1 |
| 16.07.2020 | 1 | 1 | 25.07.2020 | 3 | 1 |
| 17.07.2020 | 0 | 0 | 26.07.2020 | 0 | 0 |
| 18.07.2020 | 0 | 0 | 27.07.2020 | 0 | 0 |
| 19.07.2020 | 7 | 1 | 28.07.2020 | 3 | 1 |
| 20.07.2020 | 0 | 0 | 29.07.2020 | 1 | 1 |
| 21.07.2020 | 0 | 0 | 30.07.2020 | 1 | 1 |

S6 Table. Numbers of event pairs (0,0), (0,1), (1,0), and (1,1) for the observed binary series in 2020 and the independent series at plot LT,

| Binary variable value | Series type  Observed | Binary variable value | |
| --- | --- | --- | --- |
|  |  | 0 | 1 |
| 0 | Independent | 4 | 5 |
|  | Observed | 4.25 | 4.25 |
| 1 | Independent | 5 | 3 |
|  | Series type | 4.25 | 4.25 |
